# Transcranial Alternating Current Stimulation at Beta Frequency: Lack of Immediate Effects on Excitation and Interhemispheric Inhibition of the Human Motor Cortex

**DOI:** 10.3389/fnhum.2016.00560

**Published:** 2016-11-03

**Authors:** Viola Rjosk, Elisabeth Kaminski, Maike Hoff, Christopher Gundlach, Arno Villringer, Bernhard Sehm, Patrick Ragert

**Affiliations:** ^1^Department of Neurology, Max Planck Institute for Human Cognitive and Brain SciencesLeipzig, Germany; ^2^Institute of Psychology, University of LeipzigLeipzig, Germany; ^3^Mind and Brain Institute, Charité and Humboldt UniversityBerlin, Germany; ^4^Institute for General Kinesiology and Exercise Science, University of LeipzigLeipzig, Germany

**Keywords:** transcranial alternating current stimulation (tACS), interhemispheric inhibition (IHI), transcranial magnetic stimulation (TMS), motor cortical excitability, neuroplasticity

## Abstract

Transcranial alternating current stimulation (tACS) is a form of noninvasive brain stimulation and is capable of influencing brain oscillations and cortical networks. In humans, the endogenous oscillation frequency in sensorimotor areas peaks at 20 Hz. This beta-band typically occurs during maintenance of tonic motor output and seems to play a role in interhemispheric coordination of movements. Previous studies showed that tACS applied in specific frequency bands over primary motor cortex (M1) or the visual cortex modulates cortical excitability within the stimulated hemisphere. However, the particular impact remains controversial because effects of tACS were shown to be frequency, duration and location specific. Furthermore, the potential of tACS to modulate cortical interhemispheric processing, like interhemispheric inhibition (IHI), remains elusive. Transcranial magnetic stimulation (TMS) is a noninvasive and well-tolerated method of directly activating neurons in superficial areas of the human brain and thereby a useful tool for evaluating the functional state of motor pathways. The aim of the present study was to elucidate the immediate effect of 10 min tACS in the β-frequency band (20 Hz) over left M1 on IHI between M1s in 19 young, healthy, right-handed participants. A series of TMS measurements (motor evoked potential (MEP) size, resting motor threshold (RMT), IHI from left to right M1 and vice versa) was performed before and immediately after tACS or sham using a double-blinded, cross-over design. We did not find any significant tACS-induced modulations of intracortical excitation (as assessed by MEP size and RMT) and/or IHI. These results indicate that 10 min of 20 Hz tACS over left M1 seems incapable of modulating immediate brain activity or inhibition. Further studies are needed to elucidate potential aftereffects of 20 Hz tACS as well as frequency-specific effects of tACS on intracortical excitation and IHI.

## Introduction

Transcranial alternating current stimulation (tACS) is a form of noninvasive brain stimulation. It is known that this relatively weak sinusoidal current can influence brain oscillations and can modulate cortical networks (Fröhlich and McCormick, 2010; Ozen et al., 2010; Thut et al., 2012; Herrmann et al., 2013). However, the potential of tACS to modulate interhemispheric brain processing such as interhemispheric inhibition (IHI) remains elusive. In humans, the endogenous oscillation frequency in sensorimotor areas is an idling beta activity (13–30 Hz) peaking at 20 Hz, which typically occurs during maintenance of tonic motor output and declines during sensory information processing and active movements (Niedermeyer, 1999; Baker, 2007). Dynamics of beta-band oscillations have a bilateral activation profile and seem to play a role in interhemispheric coordination of movements and related neural activity (Houweling et al., 2010; Kilavik et al., 2013). Zaehle et al. (2010) proposed that endogenous oscillations could be enhanced by tACS at a matching frequency. Indeed, tACS applied in specific frequency bands (e.g., α, β or θ band) over primary motor cortex (M1) or the visual cortex modulates cortical excitability within the stimulated hemisphere by modulating natural brain rhythms (Zaghi et al., 2010a,b).

Transcranial magnetic stimulation (TMS) is a noninvasive and well tolerated method of directly activating neurons in superficial areas of the human brain (Barker, 1991). When delivered to the M1, TMS is a useful tool to evaluate the functional state of motor pathways (Rossini and Rossi, 2007). The cortical excitability is defined either by the resting motor threshold (RMT) or by the size of motor evoked potentials (MEPs) in the target muscle (Pascual-Leone et al., 1998; Wassermann et al., 1998; Fitzgerald et al., 2002a,b). Different TMS protocols were established to test intracortical as well as IHI/facilitation such as contralateral silent period (cSP; Cantello et al., 1992), short and long interval intracortical inhibition (SICI and LICI), intracortical facilitation (ICF; Valls-Solé et al., 1992; Kujirai et al., 1993; Wassermann et al., 1996) as well as IHI and facilitation (IHF) and ipsilateral silent period (iSP; Ferbert et al., 1992; Di Lazzaro et al., 1999; Hanajima et al., 2001a; Chen et al., 2003). Ferbert et al. (1992) first introduced the paradigm of IHI by showing that shortly after a suprathreshold conditioning TMS pulse was applied to one M1, cortical excitability of the opposite M1 decreased. IHI may play a role in motor control and in preserving hemispheric dominance, for example in bimanual coordination including suppression of undesired mirror activity (Kobayashi et al., 2003; Duque et al., 2007), since studies on patients with cortical myoclonus or patients recovering from stroke showed changes in interhemispheric interactions (Hanajima et al., 2001b; Shimizu et al., 2002).

Previous TMS-studies showed that during a short-lasting application of tACS at β-range (20 Hz) over M1, corticospinal excitability significantly increased as shown by an increase in MEP size (Feurra et al., 2011a, 2013). This effect was shown to be frequency, duration and location specific. TACS at other frequencies (5, 10, or 40 Hz) or for different periods of time as well as tACS at other stimulation sites (parietal cortex or peripheral ulnar nerve) or with different electrode set-ups influenced cortical excitability differently (Antal et al., 2008; Chaieb et al., 2011; Feurra et al., 2011a; Schutter and Hortensius, 2011). However, Cappon et al. (2016) observed reduced MEP amplitudes after 10 min of 20 Hz tACS over left M1, and Wach et al. (2013) did not find any effect of 20 Hz tACS, neither on MEP size nor on cSP of the stimulated left M1. Hence, the effects of tACS remain controversial, and knowledge concerning the underlying neurophysiological mechanisms of tACS is fragmentary. For example, as mentioned above, whether or not tACS is capable of inducing interhemispheric effects remains elusive. This knowledge could lead to a better understanding of the underlying neurophysiological mechanisms of tACS-induced neuroplastic effects within the human motor system.

Therefore, the aim of the present study was to elucidate the immediate effect of 10 min tACS in the β-frequency band (20 Hz) over left M1 on IHI between M1s in young, healthy, right-handed participants. A series of TMS measurements (MEP size, RMT, IHI from left to right M1 and vice versa) was performed before and immediately after tACS or sham using a double-blinded, cross-over design. The primary outcome measure was the immediate effect of tACS on IHI between M1s. The secondary outcome measure was the immediate effect of tACS on cortical excitability (MEP size, RMT) of both left and right M1.

Because we used stimulation parameters comparable to Cappon et al. (2016) and because, behaviorally, there is evidence that 20 Hz tACS over M1 leads to movement slowing (Pogosyan et al., 2009; Wach et al., 2013), we hypothesized that we would observe a decline in cortical excitability indicated by a decrease in MEP size and an elevation in RMT in the stimulated left M1 directly after stimulation. We further hypothesized that tACS leads to a disinhibition of the non-stimulated right M1, indicated by a reduced IHI from left to right M1 and an increased IHI from right to left M1.

## Materials and Methods

### Subjects

A total number of 19 right-handed young, healthy participants (mean age: 27.84 ± 0.82 years; range 22–35 years; 10 females) participated in the present study. All participants gave written informed consent before starting the experiment. The study was performed in accordance with the Declaration of Helsinki, and was approved by the local ethics committee of the University of Leipzig. None of the participants had a history of neurological illness, and none were taking any centrally-acting drugs during the time of the experiment. Prior to participation, all participants underwent a comprehensive neurological examination, and each participant fulfilled the inclusion criteria in agreement with the safety guidelines approved by the TMS consensus (Rossi et al., 2009). All participants were right-handed, as assessed by the Edinburgh Handedness Questionnaire (mean handedness score of 91.16 ± 3.33; Oldfield, 1971).

### Experimental Procedures

**Figure 1:**
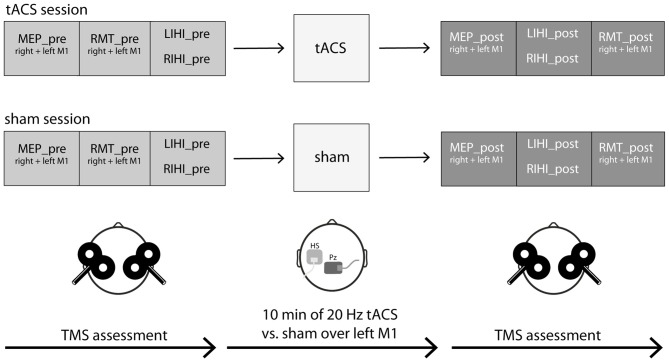
**Experimental procedures.** Nineteen young and healthy participants took part in two experimental sessions in a cross-over design on 2 days separated by at least 48 h. At each experimental session, cortical excitability (motor evoked potential (MEP) size, resting motor threshold (RMT)) and interhemispheric inhibition (IHI) were measured by transcranial magnetic stimulation (TMS) single- and paired-pulse protocols over both primary motor cortex (M1) hand areas before and after transcranial alternating current stimulation (tACS; *tACS session*) or sham (*sham session*). IHI was investigated both from left M1 to right M1 *(LIHI)* and vice versa (*RIHI*) in a randomized order. MEPs of right and left first dorsal interosseus muscle (FDI) were recorded by electromyography (EMG). The experimental sessions only differed in the type of stimulation: 10 min of 20 Hz tACS vs. sham (30 s of 20 Hz tACS) stimulation with an intensity of 1 mA each. The active electrode was placed on the FDI hotspot of the left M1, the “reference” electrode was placed on Pz according to the International 10-20 EEG system. The order of stimulation was randomized between participants and the study was performed in a double-blinded manner. Throughout the experiment, participants were seated in a comfortable chair in a relaxed position and were asked to keep their eyes open. See also “Experimental Procedures” Section for a detailed description.

Participants took part in two experimental sessions (tACS vs. sham) in a cross-over design on two separate days. To avoid any carry-over effects, each session was at least 48 h apart, and for each participant the two sessions took place at around the same time of the day. At each experimental session, cortical excitability was first assessed by measuring MEP size (*MEP_pre*) over both M1 hand areas in a randomized order between hemispheres. RMT (*RMT_pre*) of both M1 hand areas was then assessed in randomized order. Subsequently, IHI both from left to right M1 (*LIHI_pre*) and vice versa (*RIHI_pre*) was assessed in randomized order between hemispheres using TMS paired-pulse protocols. Next, 10 min of 20 Hz tACS or sham was applied over left M1. Immediately after stimulation, MEP size (*MEP_post*) of both M1 hand areas was reassessed in a randomized order. IHI (*LIHI_post* and *RIHI_post*) was then reassessed in a randomized order followed by RMT (*RMT_post*) measurements of both M1 hand areas in a randomized order between hemispheres. MEPs of right and left first dorsal interosseus muscle (FDI) were recorded by electromyography (EMG) to quantify the induced changes in excitability. The experimental sessions only differed in type of stimulation: tACS vs. sham-stimulation. The order of stimulation was randomized between participants and the study was performed in a double-blinded manner. All participants were naïve to the aim of the study (also see Figure 1). Throughout the experiment participants were asked to keep their eyes open and to relax their whole body, especially their hands, to minimize potential movement related changes in our outcome measures. Before and after each experimental session, all participants rated their levels of attention, fatigue and discomfort on a visual analog scale (VAS) to control for the effect of arousal, which may influence MEP size (Stefan et al., 2004). Additionally after each experimental session, all participants had to report whether they felt the tACS-stimulation or not to assess blinding integrity.

### EMG Recordings

EMG responses were recorded with Ag-AgCl surface cup electrodes positioned in a tendon-belly configuration over the bulk of the FDI muscle and the first metacarpal-phalangeal joint from the bilateral FDI. The signal was amplified (D360 8-channel amplifier; Digitimer, Welwyn Garden City, Herfordshire, UK) and band-pass filtered (bandwidth, 20–2000 Hz). The signal was then digitized at a frequency of 2000 Hz (CED Power1401; Cambridge Electronic Design, Cambridge, UK), fed to a data acquisition system (Signal version 4.11 for Windows, Cambridge Electronic Design) and stored on a personal computer for off-line analysis. During the experiment, the EMG was monitored on a computer screen and trials with background EMG activity were discarded from further analysis.

### Transcranial Magnetic Stimulation

For TMS, participants were seated in a comfortable chair with their hands and elbows rested on a pillow on their lap. We used two Magstim 200 stimulators, connected by a BiStim module (Magstim, Whitland, Wales, UK) and two custom-made figure-of-eight coils with 80 mm outer diameter. The coils were held tangentially to the scalp with the handles pointing backward and laterally, angled at about 45° from the midline, resulting in a posterior anterior direction of current flow in the brain. The stimulation location on both left and right M1 was determined to be the FDI hotspot where at minimum stimulator output the largest and most constant MEP in the FDI was elicited. The FDI hotspot on both hemispheres was then marked and tracked with a neuronavigation system (Brainsight 2; Rogue Research, Montreal, QC, Canada) to ensure a steady site of stimulation on both hemispheres over the experiment.

### Single-Pulse TMS Protocols

Before and after tACS or sham application, the cortical excitability of both M1s in FDI was evaluated and the minimum intensity (in % of maximum stimulator output, MSO) that could elicit a constant 1 mV MEP was defined. This intensity was then used for IHI measurements. The RMT over the FDI hotspots of both M1s was defined as the minimum stimulator output intensity that could elicit a MEP of at least 50 μV peak-to-peak amplitude in 5 out of 10 consecutive trials (Rossini et al., 1999). Both left and right M1s were assessed sequentially in a randomized order across participants before and after tACS or sham application (*MEP_pre* and *MEP_post*; *RMT_pre* and *RMT_post*). To define *MEP_pre* and *MEP_post*, the mean of 15 consecutive TMS pulses was calculated for each hemisphere.

### Interhemispheric Inhibition: Paired-Pulse TMS Protocols

A paired-pulse TMS protocol, similar to that introduced by Ferbert et al. (1992), was used to elicit IHI both from left M1 to right M1 (*LIHI*) and vice versa (*RIHI*) in a randomized order. A conditioning stimulus (CS) was delivered to M1 on one side followed by a test stimulus (TS) delivered to the contralateral M1 at an interstimulus interval (ISI) of 10 ms. The paired-pulse stimulations and TS alone were randomly applied every 5.5 s. Fifteen control MEPs (TS alone) and 15 conditioned MEPs were obtained after M1 stimulation of either left or right FDI. In total, two IHI-measurements per M1 were conducted: the first before tACS or sham (*LIHI_pre* and *RIHI_pre*), the second immediately after tACS or sham (*LIHI_post* and *RIHI_post*). The order of tested M1 was randomized between subjects and IHI measurements. The chosen intensities for TS and CS in both IHI measurements before and after tACS or sham were adjusted analogically to the study of Pal et al. (2005) to account for possible influences of CS on IHI: For *LIHI_pre* and *RIHI_pre*, TS and CS intensity was adjusted to the minimum stimulus intensity to evoke 1 mV control MEPs (as determined by initial single-pulse TMS measurements, see “Single-Pulse TMS Protocols” Section). For *LIHI_post* and *RIHI_post* the CS intensity was kept constant (as for *LIHI_pre* and *RIHI_pre*, respectively), but TS intensity was, if necessary, adjusted to evoke 1 mV control MEPs after tACS or sham.

### Transcranial Alternating Current Stimulation (tACS)

tACS was applied with a fixed frequency of 20 Hz in the β-band with an intensity of 1 mA delivered by a DC current stimulator (Neuroconn GmbH, Ilmenau, Germany). We used saline-soaked sponge electrodes to deliver the tACS and flexible elastic straps to fixate the electrodes on the head. The center of the active electrode (4.5 cm × 4.5 cm) was placed on the FDI hotspot of left M1 as determined by TMS. Electrode positioning was guided by the neuronavigation system as used for TMS. The “reference” electrode (5 cm × 7 cm) was placed on Pz according to the International 10-20 EEG system because this setting has successfully been used in previous studies (Feurra et al., 2011a, 2013). Moreover, during pilot testing, participants reported flickering sensations when the “reference” electrode was placed on the right frontal orbit, that has also been reported by Antal et al. (2008) and Wach et al. (2013). Before placement of tACS electrodes, the subject’s scalp was carefully prepared using alcohol pads in order to reduce impedance levels. During tACS or sham, impedance was always kept below 10 kΩ. At the beginning and at the end of stimulation, the current was increased in a ramp-like fashion over 30 s (Wach et al., 2013). TACS in the β-range (20 Hz) was delivered for 10 min. For sham, stimulation with 20 Hz was applied for 30 s to induce the typical tingling sensation (Zaghi et al., 2010b; Schutter and Hortensius, 2011; Wach et al., 2013). During tACS or sham, participants were asked to stay awake and relaxed in a seated position.

### Data Analyses

Statistical analyses were conducted using the Statistical Software Package for Social Sciences (IBM SPSS Version 22).

#### Primary Outcome Measure: IHI

To evaluate IHI, the amplitude of the conditioned MEPs elicited by paired-pulse stimulation were normalized by the amplitude of the control MEPs evoked by TS alone:(1 − *(mean conditioned MEP/mean unconditioned MEP)*) × 100 in %. To test for baseline differences in IHI between sessions (tACS vs. sham), a paired *t*-test was performed for each direction (*LIHI_pre* and *RIHI_pre*) and session, separately. Subsequently, a repeated-measures ANOVA (ANOVA-RM) with factor TIME (*IHI_pre* vs. *IHI_post*) and SESSION (tACS vs. sham) was performed for each direction (LIHI and RIHI) to evaluate the influence of tACS on IHI. If applicable, further *t*-tests for within and between session comparisons of IHI were performed.

#### Secondary Outcome Measure: Cortical Excitability

##### MEP

To test for differences in baseline MEP size per M1 between sessions, a paired *t*-test per M1 (left M1 and right M1) was used to compare the MEP size before tACS or sham between sessions (tACS vs. sham). To assess the effect of tACS on MEP size, an ANOVA-RM with factor TIME (*MEP_pre* vs. *MEP_post*) and SESSION (tACS vs. sham) was performed per M1 (left M1 and right M1). If applicable, further *t*-tests for within and between session comparisons of MEP size were performed.

##### RMT

*RMT_pre* between sessions (tACS vs. sham) was compared for each M1 (left M1 and right M1), separately, using a paired *t*-test to test for baseline differences in RMT. To assess the effect of tACS on RMT for each M1 (left M1 and right M1), an ANOVA-RM with factor TIME (*RMT_pre* vs. *RMT_post*) and SESSION (tACS vs. sham) was performed for each M1, separately. Subsequently, further *t*-tests for within and between session comparisons of RMT were performed, if applicable.

A Bonferroni corrected *p*-value of < 0.05 was considered to be significant and Greenhouse-Geisser correction was applied, if applicable. The Eta-squared (*η*^2^) is reported for each ANOVA as a measure of the effect size. As proposed by Miles and Shevlin (2001), we considered an *η*^2^ of ≥ 0.02 as a small, ≥0.13 medium and ≥0.26 large effect. A McNemar test was used to test for a potential difference in ratings for stimulation perceived vs. not perceived in the tACS session compared to the sham session. Behavioral data are presented as mean ± standard error (SE).

## Results

All participants tolerated the interventions without reporting any discomfort and there were no adverse events during study procedures. There were no significant differences (pre vs. post) between sessions (tACS or sham) in levels of attention (before: *t*_(36)_ = −0.076, *p* = 0.940; after: *t*_(36)_ = −0.578, *p* = 0.567), fatigue (before: *t*_(36)_ = −1.241, *p* = 0.222; after: *t*_(36)_ = −1.245, *p* = 0.221) or discomfort (before: *t*_(36)_ = −0.588, *p* = 0.560; after: *t*_(36)_ = −0.679, *p* = 0.501). Blinding integrity was assessed with the McNemar test which revealed no significant differences in the reported perception on stimulation between tACS and sham (*p* = 1.000). During tACS, nine participants perceived stimulation whereas 10 did not. Similarly during sham, eight participants perceived stimulation whereas 11 did not.

### IHI

**Figure 2:**
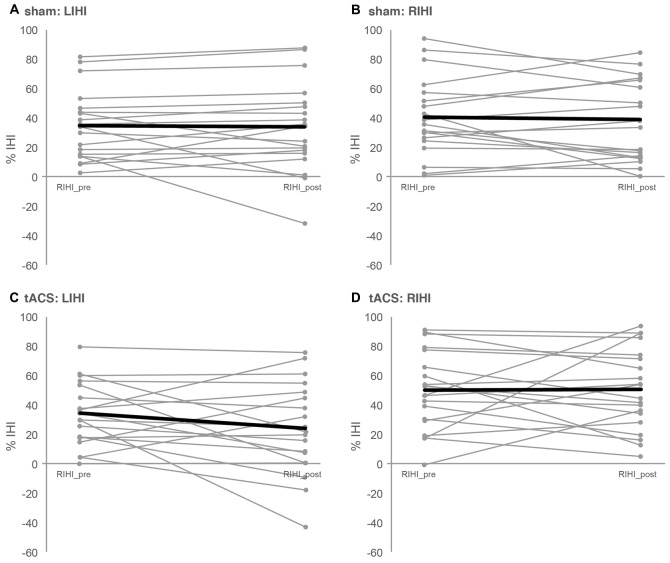
**Interhemispheric inhibition.** Depicted are the individual IHIs of each participant (slim gray lines) as well as the mean IHI (bold black lines) per session and direction of IHI measurement. IHI was assessed with paired-pulse TMS before (*pre*) and after (*post*) 10 min of 20 Hz tACS (*tACS*) or sham stimulation (*sham*). IHI from left to right M1 (*LIHI*) was obtained by delivering a conditioning stimulus (CS) to left M1 followed by a test stimulus (TS) delivered to right M1 with an interstimulus interval (ISI) of 10 ms. For RIHI, CS was applied to right M1 followed by TS applied to left M1. For *LIHI_pre* and *RIHI_pre*, the TS and CS intensity was adjusted to the minimum stimulus intensity to evoke 1 mV control MEPs. For *LIHI_post* and *RIHI_post* CS intensity was maintained, but TS intensity was adjusted, if necessary, to evoke 1 mV control MEPs after tACS or sham. **(A,B)** Depict LIHI and RIHI under sham stimulation and **(C,D)** depict LIHI and RIHI under 10 min of tACS. See “Interhemispheric Inhibition: Paired-Pulse TMS Protocols” and “IHI” Sections for details as well as Table 1.

Baseline LIHI did not differ significantly between sessions (LIHI: *t*_(18)_ = 0.214, *p* = 0.833). However; baseline RIHI differed significantly between sessions (RIHI: *t*_(18)_ = 2.659, *p* = 0.016). Ten minutes of 20 Hz tACS over left M1 did not affect IHI significantly (LIHI: ANOVA-RM with factor TIME (LIHI_pre vs. LIHI_post) × SESSION (tACS vs. sham): *F*_(1,36)_ = 1.440; *p* = 0.238; *η*^2^ = 0.038; RIHI: ANOVA-RM with factor TIME (RIHI_pre vs. RIHI_post) × SESSION (tACS vs. sham): *F*_(1,35)_ = 0.082; *p* = 0.777; *η*^2^ = 0.002). Please see Figure 2 and Table 1 for details on IHI.

**Table 1:** **Interhemispheric inhibition**.

	IHI left to right M1		IHI right to left M1	
	*LIHI_pre* (% IHI)	*LIHI_post* (% IHI)	*RIHI_pre* (% IHI)	*RIHI_post* (% IHI)
tACS	34.86 ± 4.76	24.63 ± 7.26	50.66 ± 6.21	51.11 ± 6.49
Sham	33.72 ± 5.50	32.50 ± 7.02	40.39 ± 6.12	38.61 ± 6.24

*Interhemispheric inhibition (IHI) as assessed with paired-pulse transcranial magnetic stimulation (TMS) before (pre) and after (post) 10 min of 20 Hz transcranial alternating current stimulation (tACS) or sham stimulation. To evaluate IHI, the amplitude of the conditioned motor evoked potentials (MEPs) elicited by paired-pulse stimulation were normalized by the amplitude of the control MEPs evoked by test stimulus (TS) alone. IHI was assessed from left to right M1 (*LIHI*) as well as from right to left M1 (*RIHI*) in a randomized order. Statistical analysis revealed no significant differences in baseline LIHI. However; baseline RIHI differed significantly between sessions. Furthermore, we did not find significant changes in IHI within or between sessions in neither IHI direction. Hence, 10 min of 20 Hz tACS over left M1 did not affect IHI significantly. All values are depicted as mean ± standard error of the mean. Also see “IHI” Section and Figure 2 for details of IHI*.

### MEP

Since baseline MEP size of each M1 was adjusted to about 1 mV, there were no significant differences in MEP size between sessions before tACS or sham (left M1: *t*_(18)_ = −0.731, *p* = 0.474; right M1: *t*_(18)_ = −0.766, *p* = 0.454). Furthermore, we did not find a significant change in peak-to-peak MEP amplitude between sessions (tACS vs. sham) in neither M1 (left M1: ANOVA-RM with factor TIME (MEP_pre vs. MEP_post) × SESSION (tACS vs. sham): *F*_(1,36)_ = 0.117; *p* = 0.734; *η*^2^ = 0.030; right M1: ANOVA-RM with factor TIME (MEP_pre vs. MEP_post) × SESSION (tACS vs. sham): *F*_(1,35)_ = 0.050; *p* = 0.824; *η*^2^ = 0.001). Hence, we did not find an influence of 20 Hz tACS over left M1 on MEP size. Please see Table 2 for a complete breakdown of MEP sizes before and after tACS or sham.

**Table 2:** **Motor evoked potentials**.

	Left M1		Right M1	
	*MEP_pre* (mV)	*MEP_post* (mV)	*MEP_pre* (mV)	*MEP_post* (mV)
tACS	0.83 ± 0.10	0.89 ± 0.17	1.12 ± 0.09	1.13 ± 0.14
Sham	0.90 ± 0.07	1.03 ± 0.11	1.25 ± 0.17	1.19 ± 0.18

*Peak-to-peak amplitudes of MEP of left and right primary motor cortices (M1) before (*MEP_pre*) and after (*MEP_post*) 10 min of 20 Hz tACS (*tACS*) or sham stimulation (*sham*). There was no significant difference in baseline MEP size within each M1 between sessions. Furthermore, there was no significant change in MEP size due to tACS. All values are depicted as mean ± standard error of the mean. Also see “MEP” Section for details of MEP size*.

### RMT

Over all, baseline RMT within each M1 was not significantly different between sessions (left M1: *t*_(17)_ = 1.046, *p* = 0.310; right M1: *t*_(18)_ = 0.250, *p* = 0.805). Ten minutes of 20 Hz tACS over left M1 did not affect RMT of neither M1 [left M1: ANOVA-RM with factor TIME (RMT_pre vs. RMT_post) × SESSION (tACS vs. sham): *F*_(1,35)_ = 0.731; *p* = 0.398; *η*^2^ = 0.020; right M1: ANOVA-RM with factor TIME (RMT_pre vs. RMT_post) × SESSION (tACS vs. sham): *F*_(1,35)_ = 0.074; *p* = 0.788; *η*^2^ = 0.002]; also see Table 3.

**Table 3:** **Resting motor thresholds**.

	Left M1		Right M1	
	*RMT_pre* (% MSO)	*RMT_post* (% MSO)	*RMT_pre* (% MSO)	*RMT_post* (% MSO)
tACS	41.56 ± 1.59	41.84 ± 1.53	38.74 ± 1.21	38.79 ± 1.30
Sham	40.89 ± 1.30	40.16 ± 1.38	38.58 ± 1.22	38.50 ± 1.45

*Resting motor thresholds (RMT) of left and right M1 as assessed with single-pulse TMS before (*RMT_pre*) and after (*RMT_post*) 10 min of 20 Hz tACS (*tACS*) or sham stimulation (*sham*). Note, that baseline RMT within each M1 was not significantly different between sessions and there was no significant change in RMT in neither M1 due to stimulation. % MSO, % of maximum stimulator output. All values are depicted as mean ± standard error of the mean. Please see “RMT” Section for details*.

## Discussion

The aim of the present study was to investigate the immediate effect of 10 min of 20 Hz tACS over left M1 on bidirectional IHI in young, healthy participants in a sham-controlled, cross-over design.

Baseline comparisons of MEP size and RMT of both M1 as well as bidirectional IHI (left to right M1 and vice versa) did not reveal significant differences between sessions. Contrary to our hypotheses, we did not find any significant tACS-induced modulations of intracortical excitation (as assessed by MEP size and RMT) and/or IHI. Similar null effects were observed for sham stimulation. These results indicate that 10 min of 20 Hz tACS over left M1 does not seem capable of modulating immediate brain activity/or inhibition and is therefore unlikely to induce immediate neuroplastic effects in M1.

Our findings of no effect of tACS on cortical excitability of the stimulated M1 seem to be in contrast to results of Cappon et al. (2016) who reported a decrease in MEP size after 10 min of 20 Hz tACS over left M1. However, this effect was most strongly pronounced in follow-up tests 13 min after stimulation cessation. In our study, however, TMS measurements were performed immediately after tACS. It might well be that our stimulation protocol has induced aftereffects that we were not able to detect with our experimental protocol. Hence, we cannot draw conclusions regarding potential effects of tACS on cortical excitability and IHI at earlier (i.e., online-effects) or later time points. Our rationale not to include later follow-up measurements was based on the huge body of previous studies that did not show after effects from using 20 Hz tACS (Antal et al., 2008; Feurra et al., 2011a; Schutter and Hortensius, 2011; Wach et al., 2013). However, future studies should investigate effects on cortical excitability and IHI both during and at later time points after tACS.

Another difference between our study and the one by Cappon et al. (2016) are slightly different electrode positions (left M1 and Pz in our study, left M1 and SMA in Cappon et al., 2016). Interestingly, Cappon et al. (2016) found effects of 20 Hz tACS when applied in combination with the performance of a cognitive task. Since we also applied 20 Hz tACS, but at rest, it is possible that this frequency band is primarily effective when background activity is modulated (by task activation) and not at rest. Indeed, previous study found brainstate dependent effects of tACS (Feurra et al., 2013; Neuling et al., 2013; Alagapan et al., 2016; Ruhnau et al., 2016). Therefore, it might be interesting to alter the task context to see whether effects of tACS are context-dependent and differ from effects of tACS on resting IHI. Because other studies have shown frequency-specific effects of tACS on motor function and cortical excitability (Chaieb et al., 2011; Feurra et al., 2011a; Schutter and Hortensius, 2011; Wach et al., 2013), future research might investigate the influence of tACS with different frequency bands, for example, 10 Hz on IHI. Future studies might also adapt the stimulation phase to the ongoing, individual brain oscillation and thereby the effect of stimulation, as proposed by Brittain et al. (2013).

Also, our results seem to be in conflict with those of Feurra et al. (2011a, 2013) who reported an increase in MEP size after application of 1.5 min of 20 Hz tACS. Here again, differences in electrode size and stimulation duration might contribute to these discrepancies. However, our results are in line with findings of Wach et al. (2013), who did not observe a significant effect on MEP amplitudes or cSP from 20 Hz tACS.

There are two possibilities being discussed in the literature as to how tACS might interact online with brain oscillatory activity: synchronization via entrainment and desynchronization via phase cancellation of ongoing oscillation (Brittain et al., 2013; Reato et al., 2013). To the best of our knowledge, desynchronization has only been attained with higher intensities (Brittain et al., 2013) or by longer stimulation durations of about 10–14 min (Polanía et al., 2012; Brittain et al., 2013). Hence, a modulation of interhemispheric inhibitory effects by tACS should be further investigated using different, for example, shorter or longer stimulation durations. Furthermore, tACS with higher intensities should also be investigated in future studies to shed more light on the lack of effects in our study.

Whilst the aforementioned arguments refer to potential sources of the lack of effects in the tACS protocol, one has to take into consideration how IHI was assessed in our study. Here we investigated IHI with a fixed ISI of 10 ms, whereas Ferbert et al. (1992) described a range of ISI from 6 ms to 50 ms to be associated with inhibitory effects between M1s. Further studies could investigate the effect of tACS on IHI with different ISIs and might also apply additional TMS measurements, like iSP. Chen et al. (2003) showed, that iSP and IHI evoked by the paired-pulse method at short ISIs (8–10 ms) are mediated differently and should be considered as complementary measures. Chen et al. (2003) suggested that iSP and IHI at 8 ms might be mediated through different callosal fibers and/or inhibit different neurons in the contralateral M1.

A further limitation of our study is that we cannot draw conclusions on behavioral effects of the chosen stimulation settings, because we did not assess motor performance. The fact that we did not observe tACS-induced neuroplasticity does not preclude that tACS is not sufficient to modulate behavior and or learning. In fact, a huge body of literature reported modulatory effects of tACS on behavior such as alterations in perception (Kanai et al., 2010; Feurra et al., 2011b), motor performance (Pogosyan et al., 2009; Wach et al., 2013) and cognition (Marshall et al., 2006; Sela et al., 2012).

To our knowledge, our study is the first to describe immediate effects of 20 Hz tACS on IHI. With the chosen stimulation parameters (10 min of 20 Hz tACS over left M1 with an intensity of 1 mA) and chosen TMS settings (ISI = 10 ms, inter-pulse-interval 5.5 s) we did not observe a significant effect of tACS on motor cortex excitability or IHI. Further studies are needed to elucidate the underlying neurophysiological mechanisms and to investigate potential aftereffects of 20 Hz tACS as well as frequency-specific effects of tACS on intracortical excitation and IHI.

## Author Contributions

VR, BS, CG, PR designed the experiment. VR, EK, MH performed the study. VR and PR analyzed the data and wrote the article. All authors were involved in the discussion and interpretation of the data.

## Funding

The work of EK is funded by the Fazit Stiftung GmbH, Frankfurt.

## Conflict of Interest Statement

The authors declare that the research was conducted in the absence of any commercial or financial relationships that could be construed as a potential conflict of interest.
